# *Candidatus* Neoehrlichia mikurensis and *Anaplasma phagocytophilum*: prevalences and investigations on a new transmission path in small mammals and ixodid ticks

**DOI:** 10.1186/s13071-014-0563-x

**Published:** 2014-12-04

**Authors:** Anna Obiegala, Martin Pfeffer, Kurt Pfister, Tim Tiedemann, Claudia Thiel, Anneliese Balling, Carolin Karnath, Dietlinde Woll, Cornelia Silaghi

**Affiliations:** Comparative Tropical Medicine and Parasitology, Ludwig-Maximilians-Universität München, Munich, Germany; Institute of Animal Hygiene and Veterinary Public Health, University of Leipzig, Leipzig, Germany; National Reference Center of Vector Entomology, Institute of Parasitology, University of Zürich, Zurich, Switzerland

**Keywords:** *Candidatus* Neoehrlichia mikurensis, *Anaplasma phagocytophilum*, *Ixodes ricinus*, Emerging pathogen, Small mammals, Tick-borne Pathogen

## Abstract

**Background:**

Small mammals are crucial for the life history of ixodid ticks, but their role and importance in the transmission cycle of tick-borne pathogens is mostly unknown. *Candidatus* Neoehrlichia mikurensis (CNM) and *Anaplasma phagocytophilum* are both tick-borne pathogens, and rodents are discussed to serve as main reservoir hosts for CNM but not for the latter especially in Germany. Analysing the prevalence of both pathogens in small mammals and their ticks in endemic regions may help to elucidate possible transmission paths in small mammal populations and between small mammals and ticks.

**Methods:**

In 2012 and 2013, small mammals were trapped at three different sites in Germany. DNA was extracted from different small mammal tissues, from rodent neonates, foetuses and from questing and attached ticks. DNA samples were tested for CNM and *A. phagocytophilum* by real-time PCR. Samples positive for *A. phagocytophilum* were further characterized at the *16S rRNA* gene locus.

**Results:**

CNM was detected in 28.6% of small mammals and in 2.2% of questing and 3.8% of attached ticks. Altogether 33 positive ticks were attached to 17 different hosts, while positive ticks per host ranged between one and seven. The prevalences for this pathogen differed significantly within small mammal populations comparing sites (χ^2^: 13.3987; p: 0.0004) and between sexes. Male rodents had an approximately two times higher chance of infection than females (OR: 1.9652; 95% CI: 1.32-2.92). The prevalence for CNM was 31.8% (95% CI: 22-44) in rodent foetuses and neonates (23 of 67) from positive dams, and 60% (95% CI: 35.7-80.25) of positive gravid or recently parturient rodents (9 out of 15) had at least one positive foetus or neonate. *Anaplasma phagocytophilum* was detected at a low percentage in rodents (0-5.6%) and host-attached ticks (0.5-2.9%) with no significant differences between rodent species. However, attached nymphs were significantly more often infected than attached larvae (χ^2^: 25.091; p: <0.0001).

**Conclusion:**

This study suggests that CNM is mainly a rodent-associated pathogen and provides evidence for a potential transplacental transmission in rodents. In contrast, most of the rodent species captured likely represent only accidental hosts for *A. phagocytophilum* at the investigated sites.

## Background

Small mammals play an essential role in the development of immature stages of hard ticks that mainly feed on rodents [1,2]. As wild rodents represent possible reservoir hosts for tick-borne pathogens, they may be important for the pathogens’ preservation and distribution. Belonging to the rickettsial family Anaplasmataceae, the tick-borne pathogens *Candidatus* Neoehrlichia mikurensis (CNM) and *Anaplasma phagocytophilum* are of considerable risk for human and animal health as they may cause life-threatening diseases [3,4]. *Ixodes ricinus*, the most common vector for zoonotic pathogens in Europe [5,6], is known to transmit *A. phagocytophilum* and suspected to transmit CNM to animals and humans [7,8]. However, transovarial transmission in this tick species has not been reported for either of these pathogens [7,8]. Consequently, mammalian hosts are essential for the transmission in natural life cycles of these bacteria [8-12]. CNM is an emerging pathogen first discovered as an *Ehrlichia*-like species in *I. ricinus* ticks from the Netherlands in 1999 [13]- Later, it was found in wild rats (*Rattus norvegicus*) and *I. ovatus* ticks from Japan [14]. It was observed in 11 humans [15] with immune deficiency from Europe over the last decade causing unspecific symptoms such as fever, septicaemia and weight loss [2,16,17]. Additionally, it was detected in dogs from Germany and Nigeria [18,19] and found widespread in *Ixodes* species from Europe [7,20] and Asia [14]. Wild rodent species from several Eurasian countries were investigated and found positive for this pathogen as well [9-12,21], suggesting their role as reservoir hosts. So far, CNM could not be cultivated *in vitro*, and epidemiological research on reservoir hosts is still incomplete.

*Anaplasma phagocytophilum* is an obligatory intracellular bacterium that causes granulocytic anaplasmosis in humans, dogs, horses and ruminants [8]. While reports of human granulocytic anaplasmosis in Europe are rare [8,22], *A. phagocytophilum* infections in U.S. residents are more frequent [23]. The different extent of virulence of the various *A. phagocytophilum* strains may account for this phenomenon, as the most virulent strain is most common in the USA but most probably rare in Europe [23]. Whilst wild ruminant species are expected to be reservoirs [24-26], rodent species may likewise serve as hosts [8,27]. Low prevalences for *A. phagocytophilum* were reported in rodent species such as bank voles and yellow-necked mice from Europe [28,29]. Humans and animals are therefore at risk of infection both by *A. phagocytophilum* and by CNM, however, knowledge on possible reservoir hosts, their distribution and the transmission patterns of these pathogens is still sparse. Thus, this study’s objectives were:

(i) Detection of CNM and *A. phagocytophilum* in small mammals and their ticks and comparison of their respective prevalence rates from three differently structured areas in Germany
(ii) Detection of CNM in questing ticks from two different sites over a period of five years
(iii) Evaluation of a possible transmission path for CNM in rodents

## Methods

### Study sites

Locations were identical to study sites selected in former studies by our group, in order to collect questing ticks. Traps were placed along those sites [28,30].

#### Urban area (R1)

The area “Dörnbergpark” (7.4 ha, 49°00’55.72”N, 12°05’08.89”E) is situated in the city centre of Regensburg, Bavaria, Southern Germany. It is a small park (7.4 ha), surrounded by walls, with strong anthropogenic influence which is expressed by a high frequency of visitors spending their leisure time there. The site is a well-tended park with mostly grassy landscape and only a few old trees such as oaks and maples. Large wild mammals like roe deer and wild boar are not present [31]. The park was described before [30].

#### Sylvatic area (T)

The site “Angelberger Forst” (641 ha, 48°06’36.42”N, 10°34’33.40”E), located near Tussenhausen, Bavaria, is a large forest (641 ha) with low anthropogenic influence. This mixed forest is mainly dominated by beeches, oaks and spruces. Different wild animal species are present, and the frequency of visitors is low. Therefore, there is little to no interaction between wild and domestic animals and humans [32]. A detailed description has been given before [24,30].

#### Renaturated area (S)

The third site (51°15'32.2''N, 12°21'02.5"E, 51°17'01.3''N, 12°21'00.6''E, 51°26’97.2''N, 12°32’25.6''E), located in Saxony, Eastern Germany, is part of the “Leipziger Neuseenland” (www.leipzigerneuseenland.de), a former open pit brown coal mining region near the city of Leipzig, which has been renaturated. The study site partially surrounds one of 20 artificially created lakes, called “Lake Cospuden” (436 ha). Bushes and trees less than 20 years old characterize this site. The region is a recreational area with a high frequency of visitors. Large wild animals such as wild boar and roe deer are present. The site is characterized by a sympatric existence of *I. ricinus* and *Dermacentor reticulatus* ticks and it is divided in three parts, formerly described as sites E, F and G [28].

### Sampling of small mammals and their ticks

In 2012 and 2013, small mammals were collected with Sherman© live animal traps (H. B. Sherman Traps, Inc., Tallahassee, Fla., U.S.A.) at all three study sites (official permit Site S: AZ 36.11-36.45.12/4/12-001, Site R1: 55.1-8646.4-140, Site T: 55.1-8646-2/30). Traps, baited with apple slices, were placed for at least two consecutive nights per month and site and were checked twice a day. For both Bavarian sites, 50 traps each were set up between July and October in 2012 and between April and September in 2013. In Saxony, small mammals were captured with 60 traps between March and October in 2012 and between January and September in 2013. Collected animals were anaesthetized with CO_2_ to sample blood by cardiac puncture and then euthanized by cervical dislocation and stored at −80°C. We identified all mammals using taxonomic keys [33]. Furthermore, conventional PCR targeting the *cytochrome b* gene [34] yielding an amplicon of 354 base pairs was performed to verify morphological identification for 15 randomly selected wood mice, 14 bank voles and 23 yellow-necked mice and all shrews, common voles, mouse weasels and field voles. Complete necropsy was carried out with collection of biometric data of all small mammals’ internal organs. The uteri of female gravid rodents were opened with sterile scissors. Foetuses were cut out of the amniotic sac avoiding contact with the outer surface, each with a sterile scalpel. However, contamination with maternal blood could not always be excluded. Small mammals were searched for ticks, which were stored frozen at -20°C and then identified with standard taxonomic keys [35]. From those we selected five ticks per developmental stage and per species for further analysis from at least 30 individuals per mammal species per year and site.

### Questing ticks

#### Questing ticks from former studies

A total of 2,146 questing *I. ricinus* ticks were available from former studies of our group [24, 36, Gomez Chamorro *et al*. unpublished]. Questing ticks were collected with a 1 m^2^ sized cotton flag, which was attached to a wooden stick, on a 300 m^2^ area, divided in three parts of 100 m^2^. Questing ticks were collected once in the second or third week of the month from April to June in the years 2009 to 2013 at the urban site and in the years 2011 to 2012 respectively at the sylvatic site. More details are shown in former studies [24,36]. In these past studies, ticks were morphologically identified and DNA was isolated in order to detect *A. phagocytophilum* (Table 1).

**Table 1:** **Number of questing** ***Ixodes ricinus*** **DNA samples available from earlier studies from 2009 to 2013 from site R1 and from 2011 and 2012 from site T**

**Year**	**Number of DNA samples from** ***Ixodes ricinus*** **per developmental stage / sex**								**Ticks investigated per year**
	**Site R1**				**Site T**				
	**Females**	**Males**	**Nymphs**	**Larvae** ^**d**^	**Females**	**Males**	**Nymphs**	**Larvae** ^**d**^	
2009^a^	75	83	67	-	-	-	-	-	**225**
2010^a^	60	60	60	-	-	-	-	-	**180**
2011^b^	120	120	115	-	33	46	120	145	**699**
2012^b^	115	120	120	-	109	117	120	80	**781**
2013^c^	85	88	88	-	^-^	^-^	-	-	**321**
**Total**	**455**	**471**	**450**		**141**	**174**	**300**	**225**	**2216**

^a^available from [36].

^b^available from [24].

^c^available from Gomez Chamorro (unpublished).

^d^larvae were investigated in pools of 5.

#### Questing ticks from this study

Additionally, we collected ticks by flagging at site T from April to June 2013. They were identified and processed for molecular analysis like the attached ticks (Table 2).

**Table 2:** **Number of additionally flagged questing** ***Ixodes ricinus*** **ticks collected at site T in the year 2013 together with the numbers of ticks selected for further investigation**

**Collecting month**	**Developmental stage/sex of collected** ***Ixodes ricinus*** **ticks**				**Total ticks per month**
	**Females** **N** ^**1**^ **(s)** ^**2**^	**Males** **N** ^**1**^ **(s)** ^**2**^	**Nymphs** **N** ^**1**^ **(s)** ^**2**^	**Larvae** **N** ^**1**^ **(s)** ^**2**^	
**April**	19 (18)	36 (31)	418 (30)	1(0)	**474** (79)
**May**	16 (16)	25 (25)	193 (30)	-	**234** (71)
**June**	7 (7)	18 (18)	256 (30)	-	**281** (55)
**Total of ticks per stage**	**42** (41)	**79** (74)	**867** (90)	**1**(0)	**989** (205)

^1^Number of ticks collected.

^2^Number of ticks selected for further investigation.

### DNA Extraction

Ticks were disrupted in separate tubes containing 300 μl PBS and a steel bead using the Tissue Lyser I (Qiagen, Hilden, Germany) at 20 Hz for 5 minutes. Spleen samples weighed between 0.01 and 0.05 g, depending on the small mammals’ spleen sizes. Each foetus was extracted separately in a whole piece. After addition of 300 μl lysis buffer and 30 μl proteinase K to each sample, ticks and small mammal samples were incubated overnight and blood samples (200 μl) for over 20 minutes at 56°C in a thermomixer (Eppendorf, Hamburg, Germany). DNA was extracted with the Maxwell® 16 LEV Blood DNA Kit (Promega GmbH, Mannheim, Germany) and the corresponding Maxwell® 16 System (Promega GmbH) as recommended by the manufacturer. Quantity and quality of the extracted DNA samples were determined with a spectrophotometer (Nano Drop ND-1000, Erlangen, Germany).

### PCR Methods

In order to detect CNM in small mammal and tick samples, a previously published real-time PCR targeting the *groEL* gene [7], was modified and carried out as described [9]. Tick and the small mammals’ spleen DNA were screened for CNM by real-time PCR. Blood and foetuses were also analysed from the gravid rodents whose spleen tested CNM-positive. Neonates from one positive dam were likewise analysed. A real-time PCR targeting the *msp2* gene of *A. phagocytophilum* [37,38] was performed with tick DNA in the AB-7500 Real Time PCR System (Applied Biosystems, Darmstadt, Germany) and with DNA from the small mammals’ blood, and spleen in the AB-7500 FAST Real Time PCR System (Applied Biosystems) [24]. Positive samples were further characterized at the *16S rRNA* gene locus [38-41] by using a nested-PCR as described [24]. Using the QIA quick PCR Purification Kit (Qiagen), PCR products were purified and sequenced by a commercial company (Eurofins MWG Operon, Ebersberg, Germany). Sequencing was performed with forward and reverse primers used for PCR amplification. Results were analysed with the Chromas Lite program (Technelysium Pty Ltd, South Brisbane, Australia). Sequences were aligned to available sequences in the GenBank with BLASTn (National Center for Biotechnology Information, Bethesda MD, USA)[24] and also compared to sequences obtained in earlier studies [13,25,36,39].

### Statistical analysis

Confidence intervals (95% CI) for prevalences in small mammals, questing and attached ticks were determined by the Clopper and Pearson method using the Graph Pad Software (Graph Pad Software Inc., San Diego, Ca., USA). Pearson’s chi-squared test was used with a type I error α of 0.05 to test the independence of compared prevalences. Fisher’s exact test was used for small sample sizes tested (n < 30). The Bonferroni correction was used when making multiple comparisons with Pearson's chi-squared tests. Odds ratios (OR) with 95% confidence intervals (95% CI) were computed to compare prevalences in female and male rodents.

## Results

### Trapping of small mammals

Altogether 631 small mammals of ten different species were caught of which blood (n = 443) and spleen (n = 594) samples were available (Table 3). From all sites, we collected 332 foetuses/neonates from altogether 73 dams (8 from site T, 1 from site R1 and 64 from site S). We selected 63 foetuses and 4 neonates, born in a trap, for further analysis as they derived from dams (n = 15) tested positive for CNM (Table 4).

**Table 3:** **Number, species and genders of small mammals trapped at all three study sites in 2012 and 2013**

**Rodent species**	**Number of captured rodents (f: females; m: males) per site**		
	**Site S**	**Site T**	**Site R1**
*Apodemus agrarius*	4 (3 f; 1 m)	-	-
*Apodemus flavicollis*	79 (40 f; 39 m)	99 (53 f; 46 m)	-
*Apodemus sylvaticus*	-	-	36 (8 f; 28 m)
*Microtus agrestis*	1 (m)	-	-
*Microtus arvalis*	7 (4 f; 3 m)	-	-
*Mustela nivalis*	2 (m)	-	-
*Myodes glareolus*	257 (114 f; 143 m)	139 (75 f; 64 m)	-
*Sorex araneus*	1 (f)	-	-
*Sorex coronatus*	-	5 (3 f; 2 m)	-
*Talpa europaea*	1(m)	-	-
**Total**	**352** (163 f; 189 m)	**243** (131 f; 112 m)	**36** (8 f; 28 m)

**Table 4:** **Detection of** ***Candidatus*** **Neoehrlichia mikurensis (CNM) DNA in foetuses/neonates from gravid/postgravid rodents that were positive for DNA of CNM in their spleens**

**Rodent species**	**Location**	**Dams positive for CNM in spleen/blood samples**	**Dams with at least 1 positive foetus**	**Number of positive foetuses/all foetuses (%)**
*Microtus arvalis*	S	1/1	1	4/6 (66.7)
*Microtus arvalis* ^*s*^	S	1/-	-	0/5 (0)
*Myodes glareolus*	S	1/1	-	0/5 (0)
*Myodes glareolus*	S	1/1	1	3/5 (60)
*Myodes glareolus*	S	1/1	-	0/5 (0)
*Myodes glareolus*	S	1/1	-	0/4 (0)
*Myodes glareolus* ^*s*^	S	1/-	-	0/5 (0)
*Myodes glareolus*	S	1/1	1	1/5 (20)
*Myodes glareolus*	S	1/1	1	5/5 (100)
*Myodes glareolus*	S	1/1	1	2/4 (50)
*Myodes glareolus*	S	1/1	1	4/4 (100)
*Myodes glareolus*	S	1/1	-	0/5 (0)
*Myodes glareolus*	S	1/1	1	3/4^n^ (75)
*Myodes glareolus*	T	1/1	1	3/4 (75)
*Apodemus flavicollis*	S	1/1	1	3/6 (50)
**Total**		**15/13**	**9**	**23/67 (32)**

^n^neonates; ^s^positive in spleen only.

### Ticks attached on small mammals

In total, we collected ticks from 449 out of the 631 small mammals. Apart from the field vole (*Microtus agrestis*) and the common mole (*Talpa europaea*), all other eight animal species were infested with ticks. Tick infestation rates ranged from 1 to 112 ticks with a mean infestation of 8 ticks and a median infestation of 16 ticks per small mammal. The mean infestation rates did not differ between infested male and female small mammals (χ^2^: 0.8933; p: 0.3446). With the multistage sampling strategy mentioned above, we ended up with 965 ticks of three different species (*I. ricinus, D. reticulatus, I. trianguliceps*) from altogether 186 rodents for further analysis (Table 5).

**Table 5:** **Attached ticks from captured rodents from all three sites from 2012 and 2013 and the number of CNM positives in a selected number of these ticks**

	**Developmental stage/sex of ticks per species and per location**							
**Tick species**	**Site S**		**Site T**			**Site R1**		**Total of ticks per species**
	**Nymphs** **N (p/n)**	**Larvae** **N (p/n)**	Adults* **N (p/n)**	**Nymphs** **N (p/n)**	**Larvae** **N (p/n)**	**Nymphs** **N (p/n)**	**Larvae** **N (p/n)**	
*I. ricinus*	180 (14/73)	1740 (17/377)	-	44 (1/22)	1132 (0/352)	3 (0/3)	151 (0/91)	**3250** (32/918)
*I. trianguliceps*	-	-	1 (0/1)	5 (0/5)	2 (0/1)	-	-	**8** (0/7)
*D. reticulatus*	35 (0/15)	98 (1/25)	-	-	-	-	-	**133** (1/40)
**Total**	**2053** (32/490 ^1^)		**1184** (1/381^2^)			**154** (0/94^3^)		**3391** (33/965)

(p/n): Number of ticks positive from the total number of ticks we selected for further investigation/number of selected ticks for DNA extraction and further investigation.

*female ticks.

^1^number of ticks selected from 77 rodents.

^2^number of ticks selected from 79 rodents.

^3^number of ticks selected from 30 rodents.

N: Number of ticks collected.

### Questing ticks from this study

In 2013, we collected 989 *I. ricinus* ticks at site T in total. Altogether 205 selected ticks were analysed for this study (Table 2).

### PCR analysis for CNM in small mammals

CNM was detected in four rodent species (*Microtus arvalis, Myodes glareolus, A. sylvaticus, A. flavicollis*), with prevalences ranging from 2.8% to 31.6% (Table 6). Regarding prevalences in the two main species, *My. glareolus* and *A. flavicollis*, no significant difference (χ^2^: 0.7; p: 0.4) was found. The prevalence in wood mice (*A. sylvaticus*) was significantly lower than in the two main species (*A. flavicollis* and *My. glareolus*) (χ^2^: 13.3987; p: 0.0004). Comparing the prevalence between 2012 (27.2%; 95% CI: 24-31) and 2013 (37.4%; 95% CI: 28-48), a significantly higher prevalence was observed in 2013 (χ^2^: 3.915; p: 0.048) for sites T and site S, but not for site R1. Gender distribution between *A. flavicollis* and *My. glareolus* was approximately equal (282 females; 303 males), but the prevalence of CNM in *My. glareolus* and *A. flavicollis* was significantly higher (χ^2^: 5.855; p: 0.016) in males (65.0%; 95% CI: 48-78) than in females (35.0%; 95% CI: 26-44.36). Males have a 96.5% (OR: 1.9652; 95% CI: 1.32-2.92) higher chance than females to be infected. The prevalence for CNM was 31.8% (95% CI: 22-44) in foetuses and neonates (23 of 67), from 9 out of 15 positive gravid or recently parturient rodents. These nine animals belonged to three species: *My. glareolus*, *A. flavicollis*, and *Mi. arvalis* (Table 4).

**Table 6:** **Prevalences of** ***Candidatus*** **Neoehrlichia mikurensis (CNM) in spleen samples from small mammals per site and year**

**Mammal species**	**Number of positive mammals for CNM (%)**					
	**Site S**		**Site T**		**Site R1**	
	**2012**	**2013**	**2012**	**2013**	**2012**	**2013**
*Myodes glareolus*	86/229 (38)	15/28 (54)	21/131 (16)	3/8 (37.5)	-	-
*Apodemus sylvaticus*	-	-	-	-	1/22 (4.6)	0/14 (0)
*Apodemus agrarius*	0/4 (0)	-	-	-	-	-
*Apodemus flavicollis*	28/57 (49.1)	11/22 (50)	6/84 (7.1)	5/15 (33.3)	-	-
*Sorex spp.*	0/1 (0)	-	0/1 (0)	0/4 (0)	-	-
*Mustela nivalis*	0/2 (0)	-	-	-	-	-
*Talpa europaea*	0/1 (0)	-	-	-	-	-
*Microtus arvalis*	4/7 (57.1)	-	-	-	-	-
*Microtus agrestis*	1/1 (100)	-	-	-	-	-
**Total**	**119/302** (39)	**26/50** (52)	**27/216** (13)	**8/27** (30)	**1/22** (4.6)	**0/14** (0)

### PCR analysis for CNM in attached ticks

Altogether 33 out of 965 (3.8%; 95% CI: 3-5) ticks attached to small mammals were positive for CNM (Table 5). Comparing all sites, we did not count the number of positive ticks but the number of hosts from which positive ticks were collected and the prevalence in attached *I. ricinus* ticks at site S was significantly higher (χ^2^: 14.3169; p: 0.0008) than at both Bavarian sites. Positive larvae were exclusively collected from CNM positive rodents.

### PCR analysis for CNM in questing ticks

Altogether 51 out of 2,315 (2.2%; 95% CI: 2-3) questing ticks at both Bavarian sites were positive. No significant differences were detected between sites (χ^2^: 2.1576; p: 0.1419) or between adult and sub-adult tick stages (χ^2^: 1.723; p: 0.189). There was no significant difference (χ^2^: 3.8122, p: 0.1432) at site R1 between years from 2009 to 2013 (2009: 8/225; 2010: 3/180; 2011: 7/364; 2012: 7/355; 3/261). However, there was a significant difference between the years at site T, pointing out that the highest prevalence was detected (χ^2^: 8.7623; p: 0.0125) in 2012 (17/426) compared to 2011 (4/344) and 2013 (2/203).

### Real-time PCR analysis for *A. phagocytophilum* in small mammals and attached ticks

In total, 7 out of 631 (1.1%; 95% CI: 0.5-2) small mammals were positive for *A. phagocytophilum*: 2/36 (5.6%; 95% CI: 1-19) wood mice from site R1, 1/243 (0.04%; 95% CI: 0-2.5) small mammals (bank vole) at site T, and 4/352 (1.1%; 95% CI: 0-3) small mammals (2 bank voles, 1 common vole, 1 yellow-necked mouse) at site S. *Anaplasma phagocytophilum* was neither identified in striped field mice, shrews, moles, mouse weasels nor in the only field vole. Comparing years, species and locations, no significant differences were found.

With regard to *A. phagocytophilum* in attached ticks, 16 out of 965 (1.7%; 95% CI: 1-3) were positive: 1/94 (1%; 95%CI: 0-6) *I. ricinus* (nymph) from R1, 2/374 (0.5%; 95% CI: 0-2) *I. ricinus* (larvae) from site T, and 13/450 (2.9%; 95% CI: 1.7-5) *I. ricinus* ticks (6 larvae, 7 nymphs) from site S. Nymphs were significantly more often infected (χ^2^: 25.091; p: <0.0001) than larvae. *I. trianguliceps* and *D. reticulatus* ticks tested negative for DNA of *A. phagocytophilum.*

### *Anaplasma phagocytophilum* nested-PCR and sequence analysis

Altogether 21 of the 23 real-time PCR-positive samples had a CT-value between 30 and 36.8. The nested-PCR targeting the *16S rRNA* gene was only successful for the 2 samples with a CT-value below 30. Sequencing analysis of *A. phagocytophilum* of the two attached *I. ricinus* nymphs (1 from a wood mouse at site R1; GenBank Acc.-No.: KJ769155; 1 from a bank vole from site S; Acc.-No.: KJ769156) revealed gene variant “A”, as described [40], for both.

## Discussion

### CNM in small mammals

The prevalences in rodents from our study were similar or even higher (2.8-41.2) in comparison to those from former studies from Germany (33-65%) [9], the Netherlands (0-25%) [7], France (1.8%) [1], Italy (2.9%) [10], Sweden (0-10%) [11] and Switzerland (3.9%) [42]. Site S is the only site where *Microtus* species were captured, admittedly in small numbers (n = 8) but with high prevalence for CNM (57-100%). These findings are similar to those observed in other studies from Germany (37-100%) [12] and the Netherlands (25%) [7], where *Microtus* spp. also were rarely caught in forested environments [7,12]. The lowest prevalence for CNM in rodents (2.8%) was detected at site R1, the study site with an exclusive occurrence of wood mice and the lowest number of captured individuals (n = 36) in total. The low number of captured animals may be explained by the structure of this study site, as it is a very small park with strong anthropogenic influence. Although studies from Sweden and the Netherlands [7,11] observed that wood mice are infected with CNM at a high percentage (10-22%) we found a low prevalence in this rodent species (2.8%) which is comparable to findings from another German study (0%) [12]. These divergent results suggest that prevalence rates in wood mice may depend on their living conditions. Taken together, the prevalences in *Microtus* species and the maximum range (0-100%) of prevalences for CNM in all captured species (Table 6), lead to the assumption that the infection rate of CNM depends on the rodent species. The lack of CNM detection in insectivores is in line with previous studies providing further evidence that insectivores most likely are not involved in the transmission cycle of this pathogen [7,9,11]. We found a sex-related bias (OR: 1.97) for CNM in male bank voles and yellow-necked mice. Similar findings were reported in bank voles for the rodent-borne Puumala virus (OR: 1.84) [43]. In that study, the discrepancy was explained by the males’ higher activity rate [43,44], which may be the same explanation for the bias for CNM in our study. A recent study described the lack of CNM infection in juvenile bank voles in Southern Sweden [45]. In strong contrast to this finding, we detected CNM in three of 4 new-born bank voles from a positive dam. Foetuses from 9 of 14 positive pregnant animals were positive for CNM and belonged to three different species (*Mi. arvalis, A. flavicollis, My. glareolus*) (Table 4). Although contamination with maternal blood during the necropsy cannot be fully excluded, this indicates the possibility of transplacental transmission in these rodent species and that it may well be expected as a main transmission mode in nature. However, experimental investigations are needed to prove this hypothesis.

### CNM in attached ticks

Considering that results in small mammals and attached ticks are related, as ticks may feed on the mammals’ infected blood, it is not surprising that the highest infection rate in attached ticks was observed at site S where the highest prevalence in small mammals was achieved compared to all other sites and that the prevalences in attached ticks were generally lower than in rodents. The prevalence for CNM in attached nymphs from site S was similar to the prevalence in questing nymphs and adults from a former study from this site [9]. The reason for similar results in sub-adult and adult stages could be a lack of exposure of adult ticks to CNM, as they preferably feed on larger wild mammals that apparently do not harbour CNM at a high percentage. Reports of low prevalences (6-8%) in adult ticks collected from larger mammals, such as red deer and wild boar, were already published [7]. Former studies suggested that transovarial transmission does not occur in *I. ricinus* ticks [7,9,42]. This hypothesis is supported by our study in which positive larvae derived exclusively from positive rodents, and all questing larvae were negative.

### CNM in questing ticks

While a previous study showed that prevalences in questing ticks differed over a wide range (0-45%) at site S during 2008 and 2009 [9], the infection rates at site T and site R1 (2.2%) from our study were generally low over the years. They did not differ between developmental stages, locations and the years from 2009 to 2013 at site R1. The fact that the prevalence in rodents (28.6%) is approximately ten times higher than in attached (3.8%) and questing ticks (2.2%) and that transplacental transmission in rodents is most likely, provides strong evidence that CNM is mainly a rodent-associated pathogen. A recent study also showed that small mammals transmit CNM to xenodiagnostic *I. ricinus* larvae by a high percentage (41%) which also confirmed the reservoir function of this group of mammals [42].

### PCR results for *A. phagocytophilum* in rodents

While white-footed mice, eastern chipmunks and short-tailed shrews are implicated to be reservoirs for the human pathogenic sequence type (Ap–ha) of *A. phagocytophilum* in the U.S. [46,47], there is no equivalent small mammal species reported as reservoir for this type in Europe yet. *Anaplasma phagocytophilum* was recently described to have four ecotypes with different host preferences in Europe: Ecotype I covers a wide range of different hosts and is expected to be pathogenic for humans, and to be transmitted by *I. ricinus* ticks, while ecotype II was described to be adapted to wild ungulates. Ecotype III, described to be non-pathogenic, is adapted to small mammal hosts and *I. trianguliceps* ticks [48] and ecotype IV is described to be associated with bird species but not with any other vertebrate species. Bown et al. also suggested that voles and roe deer exist in two enzootic co-existing cycles for different *A. phagocytophilum* strains [49] which was supported by two recent studies from Slovakia and Northern Italy [50,51]. Moreover, Bown et al. [27] claimed that in particular common voles could be possible reservoirs for *A. phagocytophilum* and involved in its enzootic life cycle together with *I. trianguliceps* ticks. We do not refute this statement as one out of 7 (14.3%) common voles was positive in our study. Furthermore the hypothesis of different ecotypes with a differing range of host preferences is supported by other data from our group, as we detected a high prevalence for *A. phagocytophilum* (98.9%) in wild ungulates at site T in a former study [24]. In strong contrast to this finding the prevalences in captured small mammals at this and at both of the other sites was very low (0- 5.6%) in the present study. These findings are in line with previous low prevalences in small mammals from Europe [28,29]. Therefore, our study provides no evidence for both mainly captured rodent species, *A. flavicollis* and *My. glareolus* as main reservoir hosts for *A. phagocytophilum,* but support that common voles may well be reservoirs for a certain ecotype of this pathogen. Xenodiagnostic experiments with *I. ricinus* larvae in a recent study also showed that *Apodemus* and *Myodes* species did not transmit *A. phagocytophilum* [42]. These results further support the aforementioned hypothesis.

### PCR results and gene variants for *A. phagocytophilum* in attached ticks

Detection rates for *A. phagocytophilum* were low in attached ticks from all sites and no significant differences between sites and years were noticed. The prevalences are comparable to those in rodents and to those from a recent study from Switzerland, where prevalences in attached ticks were 0% [42]. In the present study *I. ricinus* nymphs were more often infected than larvae, highlighting the possible transstadial transmission in *I. ricinus*. Infected nymphs probably fed as larvae on other vertebrate species such as hedgehogs, which may have higher infection rates of *A. phagocytophilum* [52,53]. For both positively tested nymphs, gene variant “A” [GenBank: KJ769156, KJ769156] was detected which was reported in ticks, hedgehogs, dogs, horses, a cat and a human patient before, but not in rodents and neither in ruminants [39,40,54-57]. While this gene variant was mainly reported in ticks from urban areas [52] this result is plausible for our study, as one of those positive nymphs derived from the urban site R1 and the other from the renaturated site S. Rodents were discussed to be reservoirs at site R1 [52]. In contrast to this assumption, we detected a low capture rate for rodents and a likewise low prevalence in these animals from site R1. As prevalences in questing ticks from this site were high (16.3-23%) [52], other vertebrates such as hedgehogs and birds [52,53] probably act as potential reservoirs. Moreover, the low prevalence of CNM in rodents and the higher prevalence in questing ticks from this site strengthen the assumption of other vertebrate hosts for CNM at site R1, since hedgehogs were positive for both *A. phagocytophilum* (76.1%) and CNM (2.3%) in a previous study from an urban area in Hungary [58]. The low prevalence for *A. phagocytophilum* in attached larvae further endorses the hypothesis that rodents may not be the main reservoir hosts for this pathogen, at least for certain variants.

## Conclusion

Our results strongly indicate that CNM is primarily a rodent-associated pathogen based on its high prevalence in rodents and their neonates when compared to its prevalence in ticks. CNM may be very efficiently transmitted transplacentally within rodent populations and not only via tick bites. In contrast, the low detection rate for *A. phagocytophilum* in most of all captured rodent species and host-attached ticks indicates that these rodents may serve as incidental carriers. However common voles may well be suggested as reservoirs for *A. phagocytophilum*.
